# Medical Botany

**Published:** 1827-07

**Authors:** 


*Medical Botany; or Illustrations and Descriptions of the Medicinal Plants of the London, Edinburgh, and Dublin Pharmacopœias, with those lately introduced into Medical Practice; comprising their Generic and Specific Characters; English, Provincial, and Foreign Appellations, &c. &c. &c.* By John Stephenson, M.D. Graduate of the University of Edinburgh; and James Morrs Churchill, Esq. Surgeon, Fellow of the Medico-Botanical Society of London. London, Published by Churchill, Leicester-square. Royal Octavo, Monthly Number, containing four Plates, with Letter-Press, &c.

Chemistry and Botany, the two great auxiliary sciences of Medicine, have been cultivated by Physicians in very disproportionate degrees at different periods. For more than half a century, the brilliant discoveries of Chemistry have almost eclipsed the humbler science of the vegetable world. Whether the former has occupied an undue portion of attention, is an inquiry not strictly necessary to the subject now under consideration, and one that we do not venture to determine. But that Botany has been undeservedly neglected, we mean the application of the science to the practical purposes of Medicine, and that probably from the comparatively low estimation in which it has been held, is a truth so palpable as not to require demonstration. Much of this neglect may have originated in the vain expectations once formed, of chemical remedies supplying a list of Materia Medica, so certain in their operations, so durable in their qualities, and so unchangeable in their nature, as altogether to supplant and render nugatory the perishable materials of which vegetables consist. It must be familiar to most reflecting minds, that such expectations have been carried far beyond what experience has substantiated; and we cannot, perhaps, approximate nearer the truth, than by affirming that they have eventually proved as undeserving serious consideration, as the once prevalent and absurd doctrine of signatures in plants, or the improbable and seductive though still popular notion, of there being no disease incident to man or animals, but what has its peculiar and appropriate antidote in the vegetable kingdom.

Among other causes to be assigned for the present inattention to vegetable remedies, and the scantiness of the Botanical Materia Medica, we may mention the long catalogue of reputed and traditionary virtues, sometimes of a contradictory nature, ascribed to such a vast number of plants; the uncertainty that still exists, notwithstanding all the industry and research of successive generations, in regard to many vegetables employed by the ancients; and the frequency of disappointments, arising from the wilful substitution of herbs, and ignorance, or neglect of those entrusted with the care of collecting and preparing them. To the combination of all these circumstances, operating at a period when the dawning improvements of modern chemistry promised such vast accessions to our remedial stores, we may attribute the general neglect of Medical Botany.

From the earliest ages, vegetables have been employed to alleviate human suffering, and still constitute almost the only remedies of savage nations. Among the Greeks, the writings of Theophrastus, Galen, and Dioscorides on Plants, have escaped destruction. Latin translations of the latter were very numerous in the 16th century: among the commentators upon it, the Venetian Matthiolus and the Swiss Baukin deservedly rank foremost. The laborious compilation of Pliny, and the concise treatise of Apuleus, we derive from the Latins. These authors furnish us with the information we possess respecting the vegetable remedies of the ancients. Their successors were, for the most part, but servile copyists; the Arabs, it is true, added something to the stores of their predecessors; but there was no material advancement until some time after the discoveries of Gamba and Columbus rendered the rich stores of distant countries accessible to Europe. From the invention of Printing, for a period of half a century, the *Ortus Sanitatis*—a treatise of medicinal herbs, &c. wholly compiled from preceding authors, and ornamented with wood-cuts of the rudest fabrication, was almost the only work that appeared. It went through many editions, and was translated into several languages. Brunfels printed, early in the 16th century, an Herbal, that is still esteemed for its expressive Wood engravings. A host of Writers followed soon afterwards, some of whose works, as those of Fuchius, Clusius, Cæsalpinus, &c. were original; some, as Matthiolus, were commentators on the Ancients; and others, as Dodöens, Tabernæmontamis, Gerard, &c. were compilers of herbals. All these works are still more or less valued by Botanists for their wood-cut representations of plants, and are replete with relations of their medicinal virtues: but it happens unfortunately, that in what they have borrowed from the ancients, there is often much uncertainty as to the plant; and that which is original is frequently set down upon insufficient data, especially the extremely delusive one of ascribing all recoveries from diseases to the plants which were administered.

From the Ortus Sanitatis to the middle of the last century, Herbals were common in every country. These generally consist of laborious compilations from previous writers:—They at length accumulated into such a chaotic mass as to render selection impossible, and bewilder judgment. A natural consequence was, that they became too vast for comprehension, and were altogether discarded. Succeeding writers in vain attempted to reduce these redundant compilations: the indiscriminate labours of their ancestors had so choaked the garden with weeds, that, in eradicating them, they could seldom distinguish the noxious, and could not avoid destroying the useful with the useless.

Since the large folio Herbal of Sir J. Hill, in 1745, Woodville's Medical Botany is the only work in our language that deserves notice.

Too much cannot be said in praise of the excellent coloured plates, and great variety of information it displays. Like all other works in progressive sciences, lapse of time has rendered it incomplete, and has naturally created a demand for a new undertaking to occupy the field thus opened. This, if we may judge of the future by the specimens now before us, Messrs. Stephenson and Churchill's work seems admirably calculated to supply.

With the view of directing some portion of attention to the subject under consideration, we have exceeded the limits usually devoted in this Journal to the auxiliary branches of Medicine. It must be admitted on all hands, that, whilst any efficacious vegetables are retained in our Pharmacopœias, and so many others that deserve insertion lie neglected and unknown, an acquaintance with Medical Botany ought to constitute a necessary part of professional education. Judging from the degree of attention it has attracted during the few last years, we may reasonably infer that its value and importance are already becoming more duly appreciated, and we anticipate, ere long, the revival of a branch of Medical Literature, that will contribute, in no small degree, to the improvement of the Healing Art.

Before we close these introductory observations, we would strongly impress upon the rising generation, the duty of making a practical knowledge of the Science of Botany an acquisition of their early years. Independent of its direct and immediate advantages, the beautiful simplicity observed in the arrangement of Plants, the precision in the use of terms, and the nice discrimination of the various parts of the vegetable economy, will prove valuable collateral and consecutive benefits, by the ideas of order and accuracy they will naturally instil into the mind, and by the excellent foundation they will thus prepare for future studies. Sir J. E. Smith's classical *Introduction to Botany*, and recent *English Flora*, are the best works that can be placed in the Student's hands:—They possess the advantage of being written in the vernacular tongue. Drummond's elegant *First Steps to Botany*, a work of much taste, will be found as entertaining as it is useful. The Latin *Compendium Floræ Britannicæ* will form an excellent pocket companion. A sparing use of plates—to solve doubts, not to learn species, will be advantageous, especially if access can be had to those standard works,—*Smith and Sowerby's English Botany*, or to *Curtis's* large *Flora Londinensis*, with the splendid continuation by Professor *Hooker*, of Glasgow. But, in default of these, the wood-cuts of the old Herbals may be resorted to, of which the huge folios of Gerard, edited by Johnson, 1636, and Parkinson's *Theater of Plants*, are the best.

The design of the present publication is sufficiently explained in the Title Page, which we have extracted at length: the execution is highly creditable to all parties. As regards the plates, we may observe that the colouring is, in general, excellent, and the natural habit or appearance of the Plants remarkably well depicted. After the Scientific and English Names, follow the Class and Order of the Linnæan arrangement, the Natural Order and the Generic and Specific Characters. The principal synonyms of authors, together with the Foreign and provincial appellations, come next, and then a full description of the plant, the habitat or place of its growth, and the time of flowering. This forms the strictly botanical department of the Work. Afterwards we have the Qualities and Chemical Properties, Medical and Economical Uses, Symptoms of Poisoning, Morbid Appearances and Treatment, Preparations, Officinal and Selected, collected, with much labour and ability, from a great variety of authors, and interspersed with a considerable share of original matter and observation.

We were rather surprised at not observing references to several works, that appear to us more worthy of quotation than some that are given. The plates of Sowerby and Curtis are infinitely preferable to the figures in Gerard and Parkinson. We think Woodville's Medical Botany and Stoke's Botanical Materia Medica might be usefully quoted throughout. The latter work is invaluable as an index to preceding writers; it contains many thousand references to volumes and pages, besides an extensive catalogue of their works, and must have cost the author an immense labour of research.

We purpose to devote a series of short articles to this subject, noticing the plates in each successive number of the work now lying before us, and to extract from the letter-press such parts as appear most deserving the attention of our readers, whether on account of their novelty or utility. To those who can afford it, we strongly recommend the work itself.

### NUMBER I.—JANUARY, 1827.

PLATE I. ATROPA BELLADONNA—*Deadly Nightshade or Dwale.* The natural habit of this plant is well represented in the first plate, and the colouring is good, except that the hue of the flowers is too purple, instead of being of a dull reddish brown colour. The new vegetable principle of *Atropine*, discovered by Brandes, the process for its preparation, its chemical properties, and powerful action on the animal economy, are fully noticed. Next follows a copious account of the medical properties and uses, from which we make the following extracts, as presenting what we believe to be new to most of our readers.

"As a topical remedy, the powder and decoction have been successfully applied to cancerous and ill-conditioned painful sores: and we have found sciatica, lumbago, the pain of venereal nodes, and anomalous muscular pains, readily yield to the influence of its extract, when used as a plaster. By some, a bougie, armed with it, has been applied to spasmodic strictures; and, if rubbed on the under-surface of the urethra in similar cases, it will often relieve; and likewise alleviate the pain of chordee: but, even here, its great power cannot be easily controlled; as, in some instances, the muscles of the perineum and penis have been so paralyzed for a time, that the urine has flowed away involuntarily."

It would appear, from the latter part of the above paragraph, that

Belladonna exerts a similar influence upon muscles, that it has upon the iris. Dr. Conquest writes, that in protracted labour, arising from rigidity of the os and cervix uteri, he has "seen decided benefit result from the introduction of about half a dram (drachm) of *Extractum Belladonnæ*, by gently rubbing it about the mouth and neck of the womb. It has suspended unproductive uterine action, and produced relaxation of parts, so that, on the recurrence of expulsatory pains, the os uteri has readily yielded, and permitted the head to pass."

Our limits will not allow us to notice the Symptoms of Poisoning, Morbid Appearances, and Treatment, which are given in detail from Paris, Orfila, &c. It may be useful, however, to add, that other plants are frequently used by Pharmacopolists and Druggists instead of Belladonna. We know that, in a large provincial town, the *Solanum Dulcamara, Woody Nightshade or Bittersweet*, was universally mistaken for it. An old Herbalist, being once desired to gather some for a surgeon, who found the extract he procured totally inert, produced the Black or Garden Nightshade *(Solanum Nigrum.)* Errors like these satisfactorily account for many of the contradictory statements we find recorded respecting the virtues of vegetable remedies, and forcibly illustrate the utility of a scientific knowledge of the various articles of Materia Medica. Linnæus well observed, "*primus gradus sapientiæ est res ipsas nosse.*"

PLATE II. CONVOLVULUS SEPIUM—*Great or Hedge Bindwind.* A well known and very ornamental indigenous plant, growing in most hedges and osieries, is introduced in the second plate, on account of the cathartic properties residing in its roots, as well as in most species of the same genus; Scammony and Jalap are familiar examples. The present plant deserves consideration, as a cheap and efficacious purgative.

PLATE III. LOLIUM TEMULENTUM—*Bearded or White Darnel, or Drake*, is figured, on account of the noxious effects it produces when mixed with corn. It is a grass growing abundantly in corn-fields in some countries, but, fortunately, of rather rare occurrence in Britain. Mixed with corn, either for baking or brewing, "it produces headache, vertigo, vomiting, lethargy, drunkenness, difficulty of speech, and the tongue exhibits a very strong trembling; while Seeger remarks that a trembling of the body is one of the most certain signs of poisoning by this plant." Hence the trivial name *temulentum*. We pass by numerous classical quotations from the Ancients, which prove that this plant was the *infelix lolium* of Virgil, and its noxious properties well understood. We may just observe that it is the Zirzania of the New Testament, (St. Matth. ch. xiii.) which, in our translation, is called *Tares;* and in some, as the Gaelic, *Cockle*. If the statement contained in the following passage be correct, it cannot be too widely promulgated. It certainly demands inquiry.

"We fear that Beer not unfrequently owes its powers to Darnel; being credibly informed, by an eminent practical botanist, that two acres of ground in Battersea fields were lately cultivated with it; and we know no other purpose to which it could be applied."

PLATE IV. CROTON TIGLIUM—*Purging Croton*, is said to be the only correct representation of the plant, and our authors acknowledge themselves indebted to Mr. Frost for permitting them to figure it from a drawing in the Library of the Medico-Botanical Society, as well as for much of the information contained in the letter-press.

The purgative virtues of the oil prepared from the seeds of this plant are now generally understood in this country. In its native places of growth they have been known from time immemorial, and were noticed by the botanists who first described it.

### NUMBER II.—FEBRUARY, 1827.

PLATE V. LEONTODON TARAXACUM—*Common Dandelion*. This common seed is admirably drawn and coloured. It is a well known aperient and diuretic, seldom employed, though highly esteemed, and recommended by many authors.

"The stomach is frequently irritated by its own secretions, arising from chronic inflammation affecting some of the abdominal viscera, especially the liver; and in protracted cases of this kind, where active treatment would be injurious, the decoctum Taraxaci, or the extract, administered three or four times a day, will often prove a valuable remedy. In habitual costiveness, the result of a long residence in hot climates, dandelion is a most efficient medicine; for, instead of impairing the constitution further, by producing a purgative action that it may be difficult to control, it assists the bowels in their functions, and constrains them mildly and regularly to perform them; while Dr. James Johnson ranks it among those agents that possess the power of preventing the formation of biliary concretions, by keeping up a due and healthy secretion of the liver."

The extract, prepared according to a formula furnished by Joseph Houlton, Esq. F.L.S. is recommended, as possessing *all* the virtues of the Plant. That found in the shops is often inert.

PLATE VI. DATURA STRAMONIUM—*Thorn Apple*, is well depicted. A separate figure of the ripe seeds of this plant would have been an useful addition to the plate, as cases of poisoning generally happen by children partaking of them. The testimony of Professor Bioelow is adduced, from the American Medical Botany, in support of the efficacy of the leaves smoked as tobacco in spasmodic asthma. He remarks:—"The efficacy of this medicine was called in question by Dr. Bree, who published, in the Med. and Phys. Journal, a letter, containing the result of a great number of unsuccessful trials of stramonium in asthmatic cases. It may be doubted whether any other physician has been so unfortunate in its use as Dr. Bree, since he affirms, that not one case of those under his care was benefited by it. Certain it is that, in this country, (America) the Thorn-apple is employed with very frequent success by asthmatic patients, and it would not be difficult to designate a dozen individuals, in Boston and its vicinity, who are in the habit of employing it, with unfailing relief, in the paroxysms of this distressing complaint. The cases which it is fitted to relieve are those of pure spasmodic asthma, in which it doubtless acts by its sedative and anti-spasmodic effects." We wish the American Professor had detailed the symptoms of what he designates pure spasmodic asthma more at large. We see no reason to doubt the efficacy of the remedy.

PLATE VII. represents SPIGELIA MARILANDICA—*Maryland Worm Grass, or Carolina Pink*, a plant of vermifuge powers, used by the Cherokee Indians. Our authors justly regard it as an unnecessary appendage to the Materia Medica. We think the Plate might have been spared.

PLATE VIII. ŒTHUSA CYNAPIUM—*Fool's Parsley*. This weed, occurring frequently in cultivated grounds, is sometimes mistaken for Common Parsley, and produces deleterious effects. By comparing the two Plants with this expressive figure, none of our readers will run the risk of deserving the application of its specific appellation.

NUMBER III.—MARCH, 1827.

PLATE IX. HYOSCIAMUS NIGER—*Common Henbane*. The general appearance of the plant is portrayed with great accuracy, but we think the flowers rather too small in proportion, and the beautiful veins on the corolla not sufficiently displayed. The valuable narcotic qualities of this plant are too generally known to require notice. The treatment in poisoning is the same as for other narcotics.

PLATE X. PHELLANDRIUM AQUATICUM—*Water Hemlock*, is now referred by Sir J. E. Smith, as by Lamark formerly, to the Genus Œnanthe, which it resembles in quality, as well as the structure of its fructification. The seeds are said to be carminative, diuretic, and narcotic, and have been much recommended on the Continent in pulmonary consumption. The testimonies adduced do not appear very satisfactory; but those who are disposed to make trial may administer from 15 to 30 grains for a dose. We are pretty certain that Cattle are sometimes destroyed by eating of this plant, as well as *Cicuta Virosa*.

PLATE XI. HELLEBORUS NIGER—*Black Hellebore*. This once celebrated remedy is now fallen into disuse. "Were it expunged," say the authors, "from the list of our Materia Medica, we could easily fill up the vacancy by indigenous plants of greater utility." We may, therefore, proceed to the next.

PLATE XII. LACTUCA VIROSA—*Strong-scented Lettuce.* We think the lower leaves on the stem taper too much to a point, and are too sharp posteriorly, but the general habit is exquisitely displayed. When bruised, this plant exudes abundance of milky juice, having a bitter acrid taste, from which we have no doubt a valuable substitute for opium might be prepared. Besides its known properties as a mild sedative and diuretic, it is said to allay palpitations of the heart and reduce the pulse.

"We have ascertained, to our own satisfaction, that it possesses a most important virtue, viz. *that of reducing the velocity of the pulse,* at the same time that it appears to increase its tone; and so remarkably efficient did it act on one patient, that three small doses of the tincture decreased the arterial action in the wrist, from 120 pulsations in the minute to less than 70, accompanied by intermissions. Unlike Digitalis, its effects on the brain are scarcely felt; and as the subject is one of considerable interest, and of no little consequence, we trust that our professional brethren will endeavour to elucidate our remarks by further investigations."

Here we must conclude the present notice. We trust that, whilst, on the one hand, the remarks we have made will tend to revive the study of this interesting branch of Medical Science, the copious extracts we have introduced will favourably recommend Messrs. Stephenson and Churchill's Medical Botany to the profession. The work certainly merits high commendation. It bears internal evidence of much care, extended research, great judgment, and unwearied industry, and we confidently anticipate that its reception will be such as to encourage them to proceed, instead of suffering it to remain uncompleted on account of the heavy expenses attending such publications, as has not unfrequently been the fate of similar excellent undertakings.

Nos. 4, 5 and 6 came to hand while this sheet was in the press, and, therefore, we must defer an account of them till next quarter.

---

